# Relationship between nursing students’ attitudes toward nursing profession and online learning satisfaction during COVID-19 lockdown

**DOI:** 10.1371/journal.pone.0277198

**Published:** 2022-11-03

**Authors:** Maša Černelič-Bizjak, Petra Dolenc

**Affiliations:** 1 Faculty of Health Sciences, University of Primorska, Izola, Slovenia, Europe; 2 Faculty of Education, University of Primorska, Koper, Slovenia, Europe; St John’s University, UNITED STATES

## Abstract

Formal education is crucial for the development of nurses’ professional identity and can play a decisive role in attracting students to the nursing profession. This is even more important during a public health emergency such as the COVID-19 pandemic. This study aimed to investigate nursing students’ attitudes and feelings toward their future profession and academic studies during the first COVID-19 lockdown. A cross-sectional, descriptive study was conducted on 361 nursing students. The data were collected through the Students’ attitudes toward the nursing profession during the COVID-19 outbreak scale, and the Satisfaction with online learning scale. Nursing students expressed higher levels of commitment and dedication to their profession compared to perceived job security. They were generally satisfied with their distance learning experience in terms of accessibility of study materials, adaptation of lectures and quality of communication with academic staff. However, students perceived the ICT-supported distance learning as moderately effective. Students’ satisfaction with online learning was positively related to their perceived professional commitment. In times of health crisis, faculties should consider students’ perceived quality of nursing education and attitudes toward future profession to promote appropriate professional identity.

## Introduction

The COVID-19 pandemic caused by the novel SARS-CoV-2 virus has led to a severe public health emergency of international concern. Most governments around the world imposed strict preventive measures to stop the spread of the disease, including the lockdown of public life. This also meant the temporary closure of all educational institutions. According to UNESCO global monitoring, 192 countries implemented closures by the end of March 2020, affecting almost 91% of the global learners [1]. Universities around the world have discontinued in-person teaching; student internships have also been suspended, especially those in clinical settings. The introduced distance learning and online courses delivered to students at home have raised concerns over learning quality and academic achievement [2, 3].

The global pandemic has brought new challenges, especially for nursing students, as an important part of their education involves clinical practice. Therefore, a number of studies have been conducted to examine the learning experiences of nursing students during the COVID-19 pandemic [4, 5] and the impact of online education on nursing students [6]. In a recent study, nearly half of the participating students reported lack of motivation, poor concentration, and significant learning difficulties, but praised the support of the teachers and the work of the faculty in this crisis [5]. Extensive research conducted on higher education students from ten countries has found that the impact of the online learning environment on student performance is strongly mediated by their learning satisfaction [7]. Therefore, student’s learning satisfaction is considered a reliable measure for assessing the quality of distance education [8]. Several variables have been identified to explain student learning satisfaction during the COVID-19 pandemic, including interaction, self-efficacy, student engagement, perceived teacher support, course structure [9, 10], as well as online platform usefulness, computer self-efficacy, and acceptance of online learning [11].

Moreover, it is important to note that this emergency is likely to have a significant impact on students’ attitudes and visions of their future profession as well as on their career paths. Nursing students who are about to enter a clinical internship can experience increased anxiety and fear due to the exceptional high workload and stress among health care workers during the COVID-19 pandemic [12]. Concerns about future involvement in the treatment and care of high-risk patients, insufficient knowledge about this highly contagious disease, and immense pressures on the health care system may also affect the professional identity of health care workers and nursing students [13, 14].

Given that the COVID-19 pandemic has created a new global reality, a few available studies focus on nursing students’ knowledge and attitudes toward the nursing profession during the COVID-19 emergency. A qualitative study conducted by Lovrić and colleagues [5] aimed to investigate how nursing students perceive the COVID-19 crisis in relation to their future profession. They found that most students expressed fear of infection and were afraid of the clinical environment, while recognizing their responsibility to the community and the importance of the nursing profession. The results of a Spanish study suggested that 65.3% of the nursing and medical students did not feel prepared or were poorly prepared to attend to cases of COVID-19, 38.9% stated being afraid of becoming infected, compared to 92% who indicated feeling afraid of being able to infect a family member. Despite these concerns and risks, students expressed a willingness to care for patients with COVID-19. However, students indicated a lack of knowledge about basic measures to prevent viral transmission at both hospital and community levels [15]. In a recent study, Nie and colleagues [16] investigated the professional identity of Chinese nursing students and their intention to leave the nursing profession during the COVID-19 pandemic. The results showed that students who expressed the intention to leave the nursing profession (approximately 9%) had lower professional identity scores than those who expressed the intention to stay. In addition, more knowledge about the COVID-19 disease and perception of the effectiveness of preventive and control measures contribute to a more positive professional identity among nursing students.

Therefore, it is important to investigate students’ perceptions, feelings and attitudes toward nursing profession during major public health crises, as previous studies have shown that positive attitudes are frequently found to enhance students’ interest in the subjects and their motivation for learning [17] and play an important role in the future career stability and the reduction of turnover rate [18]. Professional identity evaluation in nursing students can significantly contribute to the identification of those students who are at risk of abandoning their studies [19] and consequently taking immediate and appropriate action to support these students.

In previous research, we found no information on the association between students’ attitudes toward the nursing profession and distance learning experience during the COVID-19 pandemic. Therefore, the purpose of this study was to investigate Slovenian nursing students’ attitudes toward their future profession, in terms of commitment, dedication and perceived security of the profession, and satisfaction with online learning during the first national COVID-19 lockdown, as well as the relationship between the studied concepts.

As some studies have found (5) that most nursing students recognize their responsibility to the community and great importance of the nursing profession during health crises, we hypothesized that students would have positive attitudes toward their future profession in terms of commitment and dedication while they would have less positive attitudes toward perceived job security during the first COVID-19 outbreak. In addition, we assumed that nursing students are relatively satisfied with their online learning experience. It should be noted that in our study, distance learning was primarily related to lectures and seminars. We also assumed that nursing student’s satisfaction with online learning would be positively related to their attitudes toward their future profession.

Finally, differences in attitudes toward the profession and online learning satisfaction were examined according to students’ work experience in the health care field. We hypothesized that nursing students who are already employed in health care have lower perceptions of job security compared to students without work experience, as the former are objectively at higher risk of becoming infected with coronavirus.

Given that the research issue was related to the specific health crisis, two instruments were specifically developed for measuring nursing students’ attitudes toward their profession and satisfaction with online learning.

## Methods

### Participants

On April 2020, the invitation to take part in the research was sent to student representatives of seven nursing faculties, members of the three universities in Slovenia, Europe. The questionnaire was disseminated to undergraduate and postgraduate nursing students. The sample size was calculated based on the population of all Slovenian nursing students in the 2019/2020 academic year, totalling 2680 students. Using a 95% confidence interval with 5% margin of error, a sample size of 336 individuals was established to meet the statistical validity requirement.

The final sample consisted of 361 nursing students from six health sciences faculties, aged between 19 and 39 years (*M* = 23.92; *SD* = 5.06), of whom 82.8% were women (age: *M* = 23.54; *SD* = 4.85) and 17.2% were men (age: *M* = 25.60; *SD* = 5.63). A majority of the participants (*n =* 132; 36.6%) were in the first year of their undergraduate education, 22.4% were in their second year and 22.2% in their final professional year, while 8.6% were students who have completed studies but not yet graduated; 37 (10.3%) students from the total sample were involved in the master’s postgraduate nursing study programme. Most of the participants had a stable partner (*n* = 245, 62.7%) and lived in an urban environment (*n* = 202, 56%). At the time of the study, 33.80% (*n* = 122) of the participants were also employed in health care, thus, studying and doing clinical work (Table 1).

**Table 1:** Socio-demographic characteristic of study participants (n = 361).

Variables		n	%
**Gender**			
	Female	299	82.8
	Male	62	17.2
**Age group (years)**			
	≤ 20	90	25
	21–24	164	45.5
	24 ≤	107	29.5
**Study programme level**			
	1st	324	89.8
	2 nd	37	10.3
**Residence**			
	Rural	202	56
	Urban	156	44
**Marital status**			
	Single	135	37.4
	Partnership	226	62.6
**Employment in health care**			
	No	239	66.2
	Yes	122	33.8

### Instruments

#### Socio-demographic information

The survey included the following socio-demographic data: age, gender, level of study, marital status, place of residence, and students’ work experience in the health care (see Table 1).

#### Attitudes toward nursing profession

The Students’ attitudes toward nursing profession during the COVID-19 outbreak scale was developed on the basis of previous studies in the field of professional identity and work engagement measurement [20–22]. According to some authors, attitudes toward professional identity could be examined within the concept of work engagement, which is defined as a positive, fulfilling, work-related state [23] and often associated with terms such as involvement, commitment, passion, absorption, enthusiasm, and dedication [24]. The initial scale with 13 items has been reviewed by a team of psychologists and nursing experts with regard to item content, clarity and readability. At the end of this process, one item was dropped, and the wording of some items was slightly modified. The final 12-item scale refers to behavioural, cognitive, and emotional aspects of attitudes toward the nursing profession, and is specifically linked to the outbreak of the COVID-19 pandemic (S1 File). The scale items cover important areas of students’ commitment to the nursing profession, their view of working in health care, willingness to make future efforts in health care, consideration of a career change, perceptions of working conditions, safety and risks, and their feeling of the usefulness and importance of the nursing profession. All items are rated on a 5-point Likert scale (ranging from “strongly agree” to “strongly disagree”) with a higher score reflecting a more positive attitude toward the nursing profession. The psychometric analysis presented in the Results section revealed a three-factor structure of the scale and favorable internal consistency.

#### Satisfaction with online learning

A 6-item Satisfaction with online learning scale was designed to measure students’ satisfaction with distance learning during the COVID-19 outbreak (S1 File). The scale was created by modifying some items from multiple existing psychometrically sound instruments [8, 25]. The scale items refer to students’ experiences with academic work and learning during the COVID-19 pandemic, such as the effectiveness of distance learning and adaptation to distance learning, satisfaction with study materials, lecturer availability to student and perceived academic performance during this period. All items are rated on a 5-point Likert scale (ranging from “strongly agree” to “strongly disagree”). A higher total score reflects greater satisfaction with distance learning during the pandemic. The scale demonstrated good measurement properties (see Results section).

### Procedure and data collection

The invitation to participate in the study and the link to the online survey were sent by e-mail to the student representatives of the faculties, who forwarded the invitation to the students. Online data collection was applied to minimize any potential risks of infection and to maintain a high degree of confidentiality. Data collection was performed from April to May 2020, during the national lockdown period. The students consented to participate in the study by clicking on an embedded link and completing the electronic survey (1ka.si; https://www.1ka.si/d/en). Appropriate written information about the study’s aim and their rights as participants was added to the questionnaire. In the data collection process, a database was created that included respondents’ answers without their names, surnames and e-mail addresses to ensure anonymity. The data collection was the sole responsibility of one researcher and was maintained in a password-secured 1ka.si account. For statistical analysis purposes, the data were then exported to SPSS (S2 File).

### Ethical considerations

The study was conducted according to ethical principles [26] and in accordance with the Helsinki Declaration and the Code of Professional Ethics of Slovenian Psychologists. The participants were informed about the purpose of the study and granted their informed consent online if they were interested in participating. Confidentiality and anonymity were assured, therefore, there was no possibility to identify the participants from their responses. Approval was obtained from the institutional review board of the participating faculties before the study was conducted (number: 01/02-017/20-BMK; April 23, 2020).

### Data analysis

The data were analysed using IBM SPSS 22.0. Principal component analysis with varimax rotation was performed to assess the validity of the instruments. The Kaiser-Meyer-Olkin (KMO) measure of sampling adequacy and the Bartlett’s test of sphericity were used to determine whether the data set was appropriate for factor analysis [27]. Reliability of the instruments was measured using Cronbach’s alpha coefficient. Descriptive statistics was conducted for all the studied variables. The Pearson correlation was used to assess the associations between students’ attitudes toward nursing profession and online learning satisfaction. An independent sample *t*-test was calculated to compare the mean values of the studied variables between groups of students. A two-tailed was considered statistically significant. The level of significance was set at *p* < 0.05.

## Results

### Students’ attitudes toward nursing profession during the COVID-19 outbreak

The validity of the 12-item Students’ attitudes toward nursing profession during the COVID-19 outbreak were examined with principal component analysis factor structure of the scale. The KMO measure was high enough (0.893) and Bartlett’s test of sphericity was statistically significant (χ^2^ = 2383.05; p < 0.001) indicating that the data were adequate for factoring. Considering eigenvalues greater than one, three factors were extracted explaining 70.1% of the variance. Items 1, 2, 3, 4, 8, 9, and 10 loaded on factor 1 relating to commitment to the nursing profession, items 5, 6, and 7 loaded on factor 2 representing the perceived security, while items 11 and 12 loaded on the factor 3 which can be seen as dedication. The scale items associated with the factors and their loadings are presented in Table 2. The factor loadings were reasonably high ranging between 0.667 and 0.863.

**Table 2:** Descriptive statistics and factor loadings for the students’ attitudes toward nursing profession scale.

Subscale/item	M (SD)	F1	F2	F3
**Commitment**				
1 My satisfaction with the nursing profession has not decreased.	3.68 (1.18)	0.784		
2 I’m not worried about working in health care	3.60 (1.27)	0.746		
3 My view of work in health care is as positive as it was before.	3.60 (1.24)	0.814		
4 I’m not considering a career change from nursing to other professions.	(1.22)	0.780		
8 Despite the demanding nature of the profession, I intend to work in health care	3.58 (1.05)	0.667		
9 I’m willing to apply effort in health care work in the future.	3.91 (1.06)	0.704		
10 I intend to continue my studies in nursing.	3.89 (0.96)	0.680		
**Security**				
5 I perceive working in health care as safe enough.	2.44 (1.18)		0.799	
6 Working conditions in health care are not as alarming as people say.	2.32 (1.04)		0.773	
7 I don’t worry about getting ill or being at risk while doing my job.	2.73 (1.15)		0.758	
**Dedication**				
11 I still associate the nursing profession with a sense of usefulness and importance.	3.67 (1.20)			0.863
12 The nursing profession gives me a sense of pride, inspiration and challenge.	3.61 (1.28)			0.858
**Variance explained %**		48.5	13.6	8.0

Note: Only factor loadings greater than 0.40 appear in the table.

The Cronbach’s alpha coefficients for commitment, security, and dedication subscales, were 0.91, 0.72, and 0.85, respectively. Mean scores for the scale dimensions showed above-average expression of commitment (M = 3.78; SD = 0.92) and dedication (M = 3.64; SD = 1.16) whereas slightly below-average expression of perceived security (M = 2.50; SD = 0.90). The highest mean score was observed in item 4 –“I’m not thinking about changing the nursing profession”, where 72.9%) of the students agreed or strongly agreed with the item. Only 16.6% of the students expressed their disagreement, and thus are considering changing profession. Similarly, students largely agreed or strongly agreed with item 9 –“I’m willing to apply effort in health care work in the future” (73.9%) and item 10 –“I intend to continue my studies in nursing” (71.4% of the students). The lowest agreement was observed in items 6 –“Working conditions in health care are not as alarming as people say” and 5 –“I perceive working in health care as safe enough” with 62% and 59% of the students disagreeing or strongly disagreeing with the items, respectively.

### Satisfaction with online learning during the COVID-19 pandemic emergency

First, the validity of the 6-item scale measuring students’ online learning satisfaction during the COVID-19 pandemic was tested. The requirements for using factor analysis were met since the KMO was 0.872, and Bartlett’s test of sphericity was 962.80 (p < 0.001). As can be seen from Table 3, a one-factor solution emerged with 60.9% of variance being explained. Factor loadings for all the items were high, ranging between 0.693 and 0.837. Cronbach’s alpha was 0.87. The scale mean score was 3.41 (SD = 0.86), indicating slightly above average satisfaction with online learning among students. The lowest mean score was observed in item 1 –“I find ICT-supported distance learning currently used effective” where 42.9% of the students agreed/strongly agreed, 28.5% neither agree nor disagree, and 26.8% disagreed/strongly disagreed with the item. Moreover, the highest mean value was observed in item 4 –“I always get answers to study-related questions” with 67.3% of the students agreeing/strongly agreeing, 19.4% neither agreeing nor disagreeing, and only 11.7% disagreeing with the item.

**Table 3:** Descriptive statistics and factor loadings of the learning satisfaction scale.

Items	M (SD)	Factor loadings
1 I find ICT-supported distance learning currently used effective.	3.18 (1.11)	0.772
2 I’m satisfied with the attitude of the academic staff in the study programme.	3.46 (1.08)	0.827
3 Study materials are well prepared and accessible.	3.46 (1.07)	0.837
4 I always get answers to study-related questions.	3.71 (0.99)	0.693
5 I find distance learning well adapted to the current conditions in terms of content and time.	3.41 (1.15)	0.800
6 I rate my studies during this period as successful.	3.25 (1.19)	0.745
**Variance explained %**		60.9

### Correlations between the studied variables

Correlations between students’ attitudes toward the nursing profession and online learning satisfaction are presented in Table 4. Pearson correlation coefficients indicated a low to moderate relationship between commitment, security, and dedication subscales (p < 0.001). Learning satisfaction was weakly correlated with commitment (p = 0.023) but not with security and dedication subscales.

**Table 4:** Correlations between the attitudes toward nursing profession and learning satisfaction.

Variables	1	2	3	4
1 Attitudes–Commitment	1			
2 Attitudes–Security	0.449**	1		
3 Attitudes–Dedication	0.589**	0.244**	1	
4 Learning satisfaction	0.121*	0.095	0.074	1

Note: *—p<0.05

**—p<0.001

### Differences in the studied variables according to nursing students’ work experience in health care

The study aimed to determine the differences in the studied variables between two groups of nursing students: those not yet employed and those already employed in health care. The results of the *t*-test are presented in Table 5. Concerning students’ attitudes toward the nursing profession, the group of employed students reported significantly lower dedication scores compared to the group of not employed students.

**Table 5:** Differences in the studied variables according to nursing students’ work experience in health care.

Variables	Students	Employed students		
	M (SD)	M (SD)	t	p
1 Attitudes–Commitment	3.79 (0.88)	3.75 (0.99)	0.36	0.717
2 Attitudes–Security	2.52 (0.85)	2.45 (1.00)	0.76	0.451
3 Attitudes–Dedication	3.73 (1.11)	3.47 (1.23)	2.10	0.037
4 Learning satisfaction	3.40 (0.82)	3.43 (0.92)	-0.39	0.697

## Discussion

In order to determine nursing students’ attitudes toward the nursing profession and online learning satisfaction during the COVID-19 emergency two self-report scales were designed. The psychometric evaluation supported the factorial validity and good internal consistency of both measures.

According to our hypothesis, that nursing students have favorable attitudes toward their future profession in terms of commitment and dedication while they have less positive attitudes toward perceived job security during the first outbreak of COVID-19, was confirmed. Indeed nursing students expressed higher levels of professional commitment and mostly disagreed with the possibility of changing profession during this challenging time. This is in line with previous findings reporting that most students do not intend to leave the nursing profession due to the COVID-19 pandemic emergency [16]. Similarly, it was found out that the pandemic and the public response to nurses has positively reinforced students’ choice of a career in nursing [12]. This lower level of intention to leave the profession is associated with an increased professional commitment, and greater job satisfaction [28, 29]. According to Lee and colleague [28] professional commitment is defined as the emotional connection an individual has toward his profession. Thus, professional identity is important to nursing students and the question is what defines this commitment. One possible explanation could be that a stronger professional commitment is linked to specific personal characteristics. In this context, research has shown that nursing students are often motivated by a desire to help others [30, 31]. Nurses are often viewed as possessing not only motivation to help, but also certain personality traits related to agreeableness, such as caring for others, empathy, compassion, and altruism [32, 33]. Eley and colleagues [34] suggest that a caring nature is a principal quality of the nursing personality, and many students describe an intrinsic reward in being a nurse as a key factor in choosing nursing career.

However, it should be noted that commitment is a dynamic concept that is always the result of a perceived coherence between personality traits and the characteristics of the profession [35]. As the nursing profession has changed over time, the narrative of the good nurse remains valid [36].

Given that the nursing profession is changing in addition to or as a result of the COVID-19 pandemic, it is relevant to investigate how nursing students see their profession in the future. Most students expressed their willingness to apply efforts in health care work in the future and their intention to continue their studies in nursing despite the experience with COVID-19 pandemic and awareness of how demanding the profession can be. These findings are comparable to those of nurses demonstrating a willingness to fulfill their duty despite the heavy working conditions and many risks during the COVID-19 pandemic [37]. On the other hand, most students expressed perceiving low security in the nursing profession. Indeed, some previous studies confirmed that most students find corona viruses highly infectious and lethal [38, 39]. Safety and self-protection issues are somehow expected, as nursing students may be the victims of infection and at the same time potential carriers of the disease to the wider population. This also reflects the need to integrate specific knowledge and skills into the study programmes to prepare nursing students for similar public health emergencies. Therefore, it is important to systematically evaluate the learning needs of student nurses in extreme situations [32] such as those arising during a pandemic. In addition, universities and health care institutions worldwide need to train students in so-called disaster management, i.e. usage of nursing knowledge and skills during disaster relief to reduce life-threatening hazards and support recovery from a disaster [40].

In identifying differences in attitudes toward the nursing profession between students, it was assumed that nursing students who are already employed in health care would have lower perceptions of job security compared to students without work experience. Our results showed that employed students reported significantly lower dedication scores compared to the group of non-employed students while there was no difference between the groups in perceived job security. We would rather expect differences in the job security dimension, as students working in a hospital or other health care facility have an objectively higher risk of infection and therefore may have been more concerned. The latter was also supported by the findings of [41], demonstrating that nurses who were at the heart of the COVID-19 outbreak reported greater fear, anxiety, sadness and anger than nursing college students.

Overall, students in our study do not perceive nursing work as a safe professional activity, but they have positive attitudes toward the nursing profession it terms of commitment and dedication. Despite the current health emergency, reduced security, fear of infection and the lack of health care personnel, students still believe that they have made a good choice regarding their future profession.

In order to ensure the quality of distance education in the future and to prevent negative learning attitudes of students, it is also important to explore students’ experiences and satisfaction with online learning. Our hypothesis, that nursing students are relatively satisfied with distance learning, was confirmed. Indeed, the results of this study revealed that the students were quite satisfied with almost all segments of the educational process: accessibility of study materials, adaptation of lectures and the quality of communication with academic staff. As shown in previous studies [4, 42], the effectiveness of online learning depends on the prepared learning material, teachers’ involvement in the online environment and the interactions between teachers and students or among the students themselves. Despite the work and effort invested in online courses by teachers, the students found the ICT-supported distance learning to be moderately effective. Especially in the early stages of distance learning, when students are still getting used to new technologies and have a lack of computer skills, they may experience some difficulties with ICT equipment [43] which can be reflected in feelings of being less successful. Some research findings have highlighted learning difficulties for older nursing students living in rural areas, with work and family responsibilities and limited electronic resources [44]. This study offers valuable insight into Slovenian students’ attitudes toward the nursing profession and satisfaction with online learning during the first national COVID-19 lockdown. We found a positive correlation between learning satisfaction and professional commitment, but not with security and dedication. Although such a relationship has not been previously established in the field of nursing education, our findings can be compared with the results of previous studies showing that undergraduate students’ overall satisfaction in higher education was positively related to future employment intentions [45]. This relationship should be further explored in order to develop positive attitudes toward the nursing profession through the educational process, especially in the event of potential future health crises.

The study has some limitations that should be considered. The cross-sectional nature of the research does not allow for causal conclusions to be made about the effects of COVID-19 on the studied variables. Thus, longitudinal studies would be more appropriate to further explore the changes in attitudes toward the nursing education and profession among students. It would also be useful to investigate other aspects of students’ psychological condition (e.g., well-being, perceived stress and stress-coping strategies, social support) during this pandemic period. In further research, stratified sampling would be preferred to provide more gender-balanced samples.

### Implications for education and practice

Different sources describe the development of a professional identity in nursing as an evolving process [46, 47], with nursing education playing a key role [48]. As such, it should include specific strategies to attract students to a career in health care and encourage them to remain in this profession after graduation [49]. It is therefore extremely useful to examine nursing students’ attitudes and feelings toward their studies and future profession, which is all the more important during health crises such as the COVID-19 pandemic. The results of this study can help us better understand and manage students’ attitudes toward the nursing profession and facilitate their transition into the work environment. In particular, our findings have revealed some areas on which nursing education should focus in order to increase the safety of future health professionals and reduce the risk of infections in the workplace. Therefore, it is important that nursing faculties provide students with up-to-date education and training on COVID-19 risk factors and appropriate preventive behaviors. It would be necessary to consider a potential expansion of the current study programmes in nursing to also include students’ knowledge, skills and confidence when faced with pandemic outbreaks, especially in terms of reducing professional stress, adopting effective coping strategies, regulating negative emotional states and emphasising the importance of adequate support in the workplace. Finally, special attention should be paid to distance learning during extraordinary circumstances. Study courses should be well-organized and structured to meet the needs of all students. Moreover, faculties can provide opportunities for nursing students to improve their ICT skills and increase their computer self-efficacy beliefs. In addition, students who need it should be offered technical support to ensure successful online learning.

The findings of our study may further encourage a more systematic and in-depth research on the psychological effects of the COVID-19 emergency on the nursing student population. Namely, in this period of crisis, students’ psychological state may have an important impact on their attitudes toward their future occupation and on the formation of their professional identity. Higher education institutions should provide appropriate support and assistance to students with psychological and learning difficulties.

## Conclusion

This study has shown that nursing students demonstrate higher levels of commitment and dedication to their future profession and lower levels of perceived job security. They are generally satisfied with their distance learning experience in terms of study content and structure and the quality of perceived interpersonal relationships and communication, while they were slightly less satisfied with ICT support. Students’ satisfaction with online learning was positively related to their perceived professional commitment. The coronavirus pandemic has highlighted the vital role of existing and future health care workers as the most valuable resource for health. Therefore, more research attention should be given to nursing students and the factors that determine their professional identity development during nursing education.

## Supporting information

S1 File The students’ attitudes scale and satisfaction with online learning scale. (DOCX)

S2 File Supporting information SPSS file. (SAV)
