# Supplementary material for: Relationship between nursing students’ attitudes toward nursing profession and online learning satisfaction during COVID-19 lockdown
Source: PLoS One. 2022 Nov 3;17(11):e0277198. doi: 10.1371/journal.pone.0277198 (PMC9632773; doi:10.1371/journal.pone.0277198)
Supplement: S1 File — (DOCX) [file pone.0277198.s001.docx]

**S1 File 1: The** **Students’ attitudes toward nursing profession during the COVID-19 outbreak scale**

Guidance: Please answer according to your own true feelings due to an outbreak of a new coronavirus pandemic.

| Scale | 1-strongly disagree, 2-disagree, 3-neutral, 4-agree, 5-strongly agree | |
| --- | --- | --- |
| 1. My satisfaction with the nursing profession has not decreased. 2. I am not concerned about professional practice in nursing. 3. My view of the nursing profession is as positive as it was before. 4. I’m not thinking about changing the nursing profession. 5. Working in health care seems safe enough to me. 6. Working conditions in health care are not as alarming as people say. 7. I am not worried that I could become ill or seriously threatened. 8. Despite the difficulty of the profession, I intend to work in the nursing field. 9. I am willing to invest effort into the nursing profession in the future. 10. I have the intent to persist in the nursing study. 11. I still associate the nursing profession with a sense of usefulness and importance. 12. The nursing profession fills me with a sense of pride, inspiration, and challenge. | |  |

**Satisfaction with online learning** **during the COVID-19 pandemic emergency**

Guidance: Please answer according to your own true feelings due to an outbreak of a new coronavirus pandemic.

| Scale | 1-strongly disagree, 2-disagree, 3-neutral, 4-agree, 5-strongly agree | |
| --- | --- | --- |
| 1. The current distance learning using information and communication technology seems to me to be effective. 2. I am satisfied with the attitude of the study programme providers. 3. Study materials are well prepared and accessible. 4. I always get answers to study-related questions. 5. Distance learning seems to me to be appropriately adapted to the current situation in terms of content and time. 6. I rate my studies as successful during this period. | |  |
